# Unravelling Shared Pathways Linking Metabolic Syndrome, Mild Cognitive Impairment, Dementia, and Sarcopenia

**DOI:** 10.3390/metabo15030159

**Published:** 2025-02-27

**Authors:** Daniela Ceccarelli Ceccarelli, Sebastiano Bruno Solerte

**Affiliations:** 1 Geriatric Medicine Department, Morgagni-Pierantoni Hospital, Via Carlo Forlanini 34, 47121 Forlì, Italy; 2 Geriatric and Diabetology Unit, Department of Internal Medicine, University of Pavia, Corso Strada Nuova 63, 27100 Pavia, Italy; bruno.solerte@unipv.it

**Keywords:** metabolic syndrome, cognitive impairment, cognitive decline, Alzheimer’s disease, dementia, insulin resistance, dyslipidaemia, drug repurposing, SIRT1, sarcopenia

## Abstract

**Background**: Aging is characterized by shared cellular and molecular processes, and aging-related diseases might co-exist in a cluster of comorbidities, particularly in vulnerable individuals whose phenotype meets the criteria for frailty. Whilst the multidimensional definition of frailty is still controversial, there is an increasing understanding of the common pathways linking metabolic syndrome, cognitive decline, and sarcopenia, frequent conditions in frail elderly patients. **Methods**: We performed a systematic search in the electronic databases Cochrane Library and PubMed and included preclinical studies, cohort and observational studies, and trials. **Discussion**: Metabolic syndrome markers, such as insulin resistance and the triglyceride/HDL C ratio, correlate with early cognitive impairment. Insulin resistance is a cause of synaptic dysfunction and neurodegeneration. Conversely, fasting and fasting-mimicking agents promote neuronal resilience by enhancing mitochondrial efficiency, autophagy, and neurogenesis. Proteins acting as cellular metabolic sensors, such as SIRT1, play a pivotal role in aging, neuroprotection, and metabolic health. In AD, β-amyloid accumulation and hyperphosphorylated tau in neurofibrillary tangles can cause metabolic reprogramming in brain cells, shifting from oxidative phosphorylation to aerobic glycolysis, similar to the Warburg effect in cancer. The interrelation of metabolic syndrome, sarcopenia, and cognitive decline suggests that targeting these shared metabolic pathways could mitigate all the conditions. Pharmacological interventions, including GLP-1 receptor agonists, metformin, and SIRT 1 inducers, demonstrated neuroprotective effects in animals and some preliminary clinical models. **Conclusions**: These findings encourage further research on the prevention and treatment of neurodegenerative diseases as well as the drug-repurposing potential of molecules currently approved for diabetes, dyslipidemia, and metabolic syndrome.

## 1. Introduction

In this review, we investigate the relationship between cognitive impairment, dementia, sarcopenia, and markers of metabolic syndrome, such as altered triglycerides, the HDL-C ratio (TG/HDL-C), and insulin resistance. We outline dysregulated patterns in energy metabolism as a potential biomarker for the early diagnosis of dementia in preclinical and pre-amnestic stages, such as subjective cognitive decline (SCD). Lastly, we focus on current evidence for common pathogenetic pathways in cognitive impairment and sarcopenia, a key feature of frailty in the elderly population as well as a predictor of progression and functional dependency in patients with dementia. 

## 2. Methods

We performed a systematic search in the electronic databases Cochrane Library and PubMed. We included preclinical studies, cohort and observational studies, and trials. Case reports, editorials, conference abstracts, and non-English studies were excluded.

## 3. Discussion

Insulin resistance, a feature of metabolic aging, is characterized by elevated fasting blood insulin and glucose levels and has been associated with a decline in cognitive function. Impaired neuronal glucose metabolism is particularly evident in the temporal, parietal, and frontal lobes. Impaired fasting glucose, formerly known as prediabetes, is a risk factor for neurodegeneration and dementia, independent of the progression to diabetes [1].

Insulin binding to its membrane receptor, a tyrosine kinase receptor, leads to the phosphorylation of protein IRS-1. Insulin resistance is characterized by reduced tyrosine phosphorylation of IRS-1 and increased phosphorylation on serine 312 of IRS-1 [2]. This increase in serine 312 IRS-1 phosphorylation has been demonstrated in post-mortem brain tissue from elderly patients, suggesting neuronal insulin resistance. Glucose metabolism pathways in AD are often impaired, whereas the utilization of ketone bodies, such as β-hydroxybutyrate and acetoacetate, is preserved.

Animal models fed on high-fat or high-sugar diets show hallmarks of brain aging like oxidative damage, neuroinflammation, impaired neuronal calcium homeostasis, and impaired autophagy [3]. Insulin resistance and obesity induced by diet or genetic mutation of the leptin receptor in mice leads to a decrease in synaptic density of hippocampal dentate granule neurons and compromised hippocampal synaptic plasticity [4]. These neural impairments coincided with decreased levels of brain-derived neurotrophic factor (BDNF) in the hippocampus. Moreover, high-fat diet consumption has been linked to decreased expression of proteins essential for neurotransmitter release, such as the 25 kDa synaptosomal-associated protein (SNAP-25). Altered levels of these proteins affect synaptic plasticity. Both preclinical and clinical studies point out that chronic positive energy balance negatively affects brain structure and function. High-fat diets lead to neuroinflammation, overexpression of inflammatory cytokines, increased activation of the mammalian target of rapamycin (mTOR), and dysregulation of AMP kinase, a cell energy sensor that plays a role in memory formation [5]. Excessive energy intake has been associated with a reduced expression of BDNF, PGC1α, and SIRT1, with a compound pathological effect on cognitive performances, as well as carcinogenesis [6].

Sirtuins are key regulators in cellular aging and energy. Sirtuins are a family of class III histone deacetylases [7] acting as energy sensors and regulators of nutrients and energy metabolism in response to modifications in diet and stress. Sirtuins require NAD^+^ as a cofactor for their enzymatic activity. Energy deficits induced by calorie restriction or physical exercise, oxidative stress, hypoxia, DNA damage, genotoxic stress, low-glucose and low-amino acid states, and mitochondrial stress might all increase NAD^+^ levels and activate sirtuins [8]. Sirtuins play a key role in DNA repair, inflammation, and stress resistance. SIRT1, currently the most known sirtuin homolog, is expressed in metabolically active tissues such as the liver, skeletal muscle, adipose tissue, pancreas, and brain. SIRT1 regulates β cell and neuron survival, hepatic gluconeogenesis, insulin secretion, and adiposity [9]. 

SIRT1 acts on many substrates, including the transcription factors fork head box O (FOXO) 1, 3, and 4, the nuclear factor kB (NF-kB) subunit p65, and the peroxisome proliferator-activated receptor γ (PPARγ) coactivator 1α (PGC-1α). Other important substrates of SIRT1 include histones H1, H3, and H4, and histone deacetylation regulates gene silencing. SIRT1 has been associated with remarkable neuroprotective effects. In the mitochondria, SIRT1 regulates the activity of PGC-1α, affecting cellular health span and longevity. SIRT1 inhibits NF-kB, a transcription factor NF-kB promoting inflammatory response [10]. In AD, there is a buildup of β-amyloid peptides, and this has been shown to be associated with activation of the NF-kB inflammatory pathway in surrounding glia.

Resveratrol is a phytoalexin, a non-flavonoid polyphenolic compound found in some plants, such as grapes, and in red wine; it is an SIRT1 activator. Resveratrol supplementation causes the inhibition of NF-kB via deacetylation pathways regulated by SIRT1, thus diminishing amyloid burden and neurotoxicity in vitro in preclinical animal models and in preliminary clinical studies [11]. SIRT1 and sirtuin-activating compounds [12] may have neuroprotective effects in AD and other related neurodegenerative conditions by inhibiting the inflammatory effect of NF-kB.

A further feature of the aging brain is dysfunctional autophagy, in particular, in oligodendrocytes. SIRT1/AMPK signaling pathways have been shown to play a key role in autophagy by inducing mitochondrial fragmentation and slowing neurodegenerative progression [13]. Experiments have shown that the activation of AMPK increases the intracellular NAD+/NADH ratio, leading to enhanced activity of SIRT1. Hence, the SIRT1/AMPK axis is increasingly being considered as a potential therapeutic target in neurodegenerative diseases where autophagy and mitophagy are dysfunctional. Overall SIRT1 activity led to a decreased rate of p53-induced cell death and eventual neurodegeneration seen in diseases, such as AD, and other neurodegenerative conditions. 

**Figure 1:**
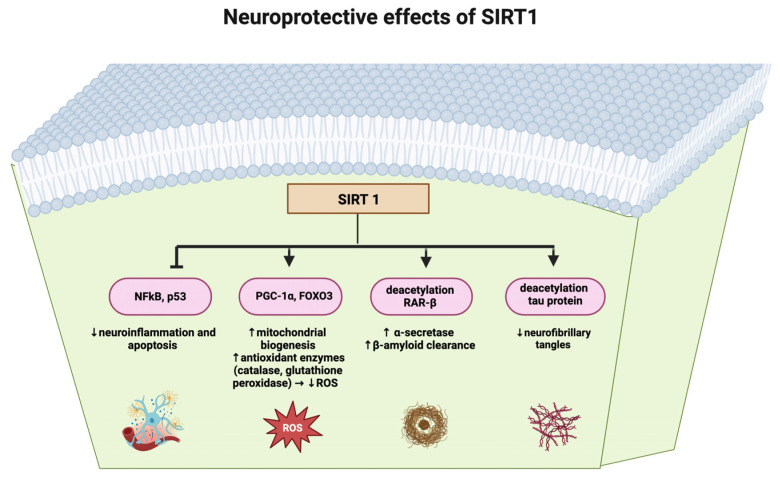
Overview of neuroprotective pathways activated by SIRT1.

AD is a neurodegenerative disorder characterized by the formation of neuritic senile plaques as well as neurofibrillary tangles. These both contribute to neuronal death due to toxicity. Recent studies suggest that the activity of SIRT1 may be involved in the interference of processes that produce both neuritic senile plaques and neurofibrillary tangles [14]. Studies show that calorie restriction causes the activation of SIRT1, which then promotes AD neuroprotection by changing transcription factor activity. Neuroprotective pathways of SIRT1 are summarized in Figure 1.

Several pharmacological agents have been studied for their effects on SIRT1; resveratrol and cilostazol are two SIRT1 activators that have been explored in depth. Resveratrol is a polyphenol and antioxidant found in the skin of red grapes, berries, and peanuts. Its potential benefits have also been evaluated for cardiovascular health and antitumoral activity. Cilostazol is a phosphodiesterase-3 inhibitor primarily used as a vasodilator and antiplatelet agent. It is approved for treating peripheral artery disease.

Some of the many other compounds, nutraceutical and pharmacological, with demonstrated SIRT 1 activator properties include metformin, Olmesartan, SRT 2104, methylene blue, curcumin, quercetin, nicotinamide riboside, and pterostilbene.

Metformin directly activates SIRT1, leading to increased deacetylation of downstream targets, such as PGC-1α, promoting mitochondrial biogenesis, and improving insulin sensitivity. Metformin also modulates AMPK, further potentiating SIRT1 activity indirectly [15]. Olmesartan induces SIRT1 mRNA expression by inhibiting the angiotensin II type 1 receptor, which reduces reactive oxygen species and inflammation [16]. SRT 2104 is an allosteric activator of SIRT1 that binds directly to an allosteric site on the enzyme, enhancing its catalytic efficiency. This interaction increases the affinity of SIRT1 for acetylated lysine residues on target proteins such as p53, NF-kB, and PGC-1α. SRT2104 promotes the deacetylation of these substrates without altering NAD+ levels [17]. Methylene blue activates SIRT1 by acting as an electron donor in the mitochondrial electron transport chain, leading to increased ATP production and decreased ROS levels. This enhances AMPK activation, which in turn upregulates NAD+ biosynthesis and SIRT1 activation, an effect that has shown beneficial effects even on hepatic steatosis [18]. Curcumin indirectly activates SIRT1 by scavenging ROS and inhibiting NFκB; curcumin also binds directly to the SIRT1 active site, increasing the deacetylation of mitochondrial proteins involved in oxidative phosphorylation [19].

Quercetin activates SIRT1 through the AMPK pathway by reducing ROS and promoting mitochondrial biogenesis. Quercetin also inhibits mTOR and activates AMPK, leading to higher NAD+ levels [20].

Nicotinamide riboside serves as a precursor for NAD+ biosynthesis through the Preiss–Handler and salvage pathways; it is a precursor of nicotinamide mononucleotide, which is subsequently converted to NAD+ [21].

Pterostilbene activates SIRT1 by directly binding to the enzyme and enhancing its deacetylase activity. Additionally, it increases cellular NAD+ levels and inhibits NAD-consuming enzymes, such as CD38. Pterostilbene-mediated SIRT1 activation promotes the deacetylation of tau proteins and suppresses amyloid β aggregation in neurodegenerative models [22].

The activation of SIRT1 by resveratrol has been found to prevent Aβ-induced microglial death, which contributes to improved cognitive function [23].

Moussa et al. performed a retrospective analysis on AD patients treated with oral resveratrol or a placebo for 52 weeks. The group found that patients treated with a placebo had a two-fold greater decline in MMSE scores, while those treated with resveratrol had a significant decrease in metalloproteinase-9 (MMP9) levels in the CSF. This study is limited by a small sample population (119 participants) [24]. Table 1 provides a summary of compounds with SIRT1 inducer activity, mechanism of action, and main effects.

SIRT1 has been strongly associated not only with neuroprotection but also with the amelioration of insulin resistance by silencing the expression of the protein tyrosine phosphatase 1B, a major negative regulator of insulin action. In recent years, several negative regulators of insulin action that contribute to insulin resistance in obesity and type 2 diabetes have been identified, including protein tyrosine phosphatase 1B (PTP1B). PTP1B is a ubiquitously expressed phosphatase that regulates several growth factor signaling pathways in vivo. PTP1B negatively regulates insulin action directly as an insulin receptor phosphatase by promoting adiposity through the negative regulation of leptin action on body weight and energy expenditure [28]. 

A direct regulation of insulin action by SIRT1 through its actions is used to control PTP1B gene expression. Thus, SIRT1 improves insulin sensitivity under insulin-resistant circumstances by silencing PTP1B expression. PTP1B deficiency in neurons promotes leanness, prevents diet-induced obesity, and enhances insulin sensitivity [29]. In addition to its role in neuroprotection, resveratrol might restore insulin sensitivity partly due to SIRT1 silencing of neuronal PTP1B expression. 

The therapeutic potential of SIRT1 inducers presents a compelling avenue for the treatment of age-related and metabolic diseases. However, the current body of evidence is derived primarily from preclinical studies and small-scale clinical trials, which limits the generalizability and robustness of the findings. To fully elucidate the efficacy and safety of SIRT1 activators, there is a need for larger, multicenter trials that encompass diverse patient populations. Such investigations will provide critical insights into the long-term effects, potential adverse events, and overall therapeutic index of these agents, thereby guiding their integration into clinical practice with a more comprehensive risk-benefit profile.

A further intervention that showed significant neuroprotective potential is intermittent energy restriction, which improves cognitive and motor performance and can protect neurons against dysfunction and degeneration in animal models of different central nervous system pathologies, including AD [30].

Even the depletion of liver glycogen stores and the mobilization of fatty acids from adipose cells have neuroprotective consequences [31]. The ketone body β-hydroxybutyrate (BHB) acts as a signaling molecule for BDNF gene expression by activating the transcription factor NF-kB [32]. Similar to myocytes, it seems that neurons react to intermittent energy restriction and exercise by improving their resilience to metabolic stress and growth. 

Intermittent metabolic switching enhances mitochondrial efficiency in brain tissue, in part by inducing the expression of the mitochondrial protein deacetylase SIRT3, leading to enhanced antioxidant activity of the enzyme SOD2, preventing excitotoxicity and apoptosis [33]. These kinds of “metabolic challenges” also temporarily suppress mTOR and overall protein synthesis, with upregulation in autophagy. When normal feeding and energy intake are restored, mTOR is activated and protein synthesis is increased, resulting in neurite growth, neurogenesis, and synaptogenesis [34].

Pharmacological approaches that tap those pathways engaged by fasting and exercise, acting as “fasting mimic”, are being investigated for the purposes of neuroprotection. The administration of the NAD+ precursor nicotinamide riboside, enhancing the mitochondrial function and SIRT3 activity, has been demonstrated to be beneficial in AD models [25,26,27]. A similar attempt has been pursued with molecules that induce mild intermittent bioenergetic cellular stress and with mitochondrial uncoupling agents, such as ATP-dependent K+ channel openers or 2-deoxyglucose [35]. These compounds disrupt the proton gradient across the mitochondrial membrane, leading to a controlled decrease in ATP production efficiency. This uncoupling effect can reduce the production of reactive oxygen species (ROS) and promote metabolic flexibility. The glucose analog 2-deoxyglucose inhibits glycolysis by blocking hexokinase activity, leading to reduced ATP production and mimicking a state of energy deprivation, resulting in mild metabolic stress and adaptive cellular responses, including autophagy and enhanced mitochondrial function. 2-Deoxyglucose has been studied not only for its neuroprotective potential but also for its ability to sensitize cancer cells to therapy and as a caloric restriction mimetic.

Other examples of therapeutic interventions targeting insulin resistance are the insulin-sensitizing hormone glucagon-like peptide 1 (GLP1) and metformin. GLP-1 receptors are coupled to cyclic AMP production and CREB activation. GLP-1 receptor agonists increase insulin sensitivity and are neuroprotective in animal models of AD. The Evaluating Liraglutide in Alzheimer’s Disease (ELAD) trial, a double-blind, placebo-controlled intervention on 204 patients with mild Alzheimer’s disease, found that those treated with the GLP1 Liraglutide experienced a slower decline in cognitive function and reduced brain atrophy compared to the placebo group [36].

A review published in the Journal of Biomedical Science explored the potential of GLP-1 receptor agonists as therapeutic agents for neurodegenerative diseases, including Alzheimer’s and dementia. Both preclinical and clinical studies suggest that these agents may provide benefits beyond glycaemic control, such as neuroprotection. GLP-1 receptor agonists have been shown to decrease the accumulation of amyloid-beta plaques and tau-related neurofibrillary tangles, promote synaptic plasticity, and bolster mitochondrial function and integrity in neurons and apoptotic pathways that lead to cell death [37].

A study published in Alzheimer’s & Dementia analyzed the electronic medical records of over one million patients with type 2 diabetes. The findings suggested that the GLP-1 receptor agonist semaglutide was associated with a 40% to 70% reduced risk for first-time AD diagnosis, most strongly compared with insulin and most weakly compared with other GLP-1 receptor agonists. The results were similar regardless of obesity status, gender, and age group [38].

Metformin treatment ameliorates neurodegeneration and behavioral phenotypes in animal models of AD. While animal studies suggest potential neuroprotective effects, human observational studies have shown conflicting results regarding metformin’s impact on cognitive decline and dementia risk [39].

Although further evidence is required to confirm these clinical benefits in clinical settings and to support the use of these molecules in the prevention or treatment of cognitive impairment, these findings support a “repurposing potential” of pharmacological agents that target metabolism to tackle brain aging processes.

Diabetes is associated with a 25–91% risk of developing dementia, whilst metabolic syndrome increases the risk of progression from mild cognitive impairment (MCI) to dementia. In a meta-analysis carried out on nine longitudinal studies with 18,313 participants, there was no significant association between metabolic syndrome and incident dementia or Alzheimer’s disease (AD). Nevertheless, metabolic syndrome affected the incidence of vascular dementia [40]. 

One of the markers of metabolic syndrome is dyslipidemia; in particular, high triglycerides and the HDL-C ratio (TG/HDL-C) are known risks of coronary heart disease. High TG/HDL-C has been associated with insulin resistance and low-grade systemic inflammation [41]. There is no similarly solid evidence on the role of TG/HDL-C on Alzheimer’s disease incidence and progression [42,43].

Nagga et al. demonstrated that increased levels of triglycerides in middle-aged subjects predict brain β-amyloid and tau pathology up to 20 years later in cognitively healthy individuals, suggesting an involvement of lipid imbalances in the very early stages of AD [44].

Increased plasma HDL levels have been associated with higher cognitive performance in older populations, with low HDL representing a risk factor for cognitive decline in adults. Apolipoprotein A1 (ApoA1) is a regulator of cholesterol efflux. Therefore, *APOA1* gene polymorphism might foresee future cognitive impairment. Lower plasma ApoA1 and HDL levels correlate with a higher amyloid burden, an increased risk for AD [45], and increased cognitive score severity in AD. ApoA1 has also a role in regulating the immune system and myeloperoxidase (MPO) activity, an enzyme that also influences neutrophil granulocytes and immune efficiency. In animal models, altered MPO levels in AD affect disease progression. Then, we speculate that in the future, attention to ApoA1 polymorphism, alongside the known allele variant Apo E ε4, might increase the predictivity for AD onset and the cluster of pathologies, such as AD metabolic syndrome or AD dyslipidemia. 

A possible explanation of the influence of cholesterol metabolism on β-amyloid deposition lies in the fact that cholesterol is locally synthesized in the brain and that there is a minimal flow of HDL-C and no flow of LDL-C across a healthy blood–brain barrier [46]. In transgenic APP mice, a high-cholesterol diet was associated with greater β-amyloid deposition. Moreover, β-amyloid proteostasis appears to be impaired by excessive cholesterol levels within the brain. There is evidence that systemic hyperlipidemia may damage the blood–brain barrier with the passage of serum cholesterol and inflammatory cytokines. The amyloidogenic role of altered cholesterol levels, both in the brain and systematically, might also be affected by dietary patterns; Galloway et al. noticed that a high-fat diet affects the conformational state of APO E, reducing its clearance ability for β-amyloids [47].

These data might support the hypothesis of the role of statin treatment in more effective β-amyloid clearance, an effect that has been observed already in vitro. To date, there are no consistent clinical trials reporting a clear benefit of statins in ameliorating cognitive decline. Data from cohort studies and meta-analysis highlighted a promising opportunity in statins repurposing for AD prevention and co-treatment. Petek et al. analyzed data from 15,586 patients with Alzheimer’s disease or mixed dementia; in this cohort study, statin use was associated with a slower cognitive decline and was measured by mini-mental status examination compared to patients who were not on statin treatment [48].

A meta-analysis by Wong et al. suggested that statins may provide a slight benefit in the prevention of AD and all-type dementia but pointed out that their results should be interpreted with caution, as observational studies are subject to bias, and this preliminary evidence should be confirmed in randomized controlled trials [49]. 

A Cochrane review in 2016 evaluated the efficacy and safety of statins for the prevention of dementia; no evidence was found to support the use of statins in preventing cognitive decline or dementia in patients without a more suitable indication, such as hypercholesterolemia or secondary prevention from cardiovascular events. Despite a lack of current evidence supporting the use of lipid-lowering drugs in AD or other dementia, plasma levels of ApoA1 and TG/HDL-C appear to be a promising prognostic marker for the rate of cognitive decline in MCI and AD dementia subjects, and their therapeutic modification by diet and medication is likely to be an interesting opportunity in the near future [50].

AD is characterized by a pattern of progressive hypometabolism on fluorodeoxyglucose positron emission tomography (FDG-PET) scans. Peripheral insulin resistance increases AD risk even without hyperglycemia and for serum glycaemic levels far lower than the threshold for diabetes diagnosis. In a study enrolling 26 cognitively normal patients, 194 MCI patients, and 60 patients with AD, HOMA-IR was used as a measure of insulin resistance. For AD, higher HOMA-IR predicted lower FDG uptake in PET scans. For MCI identified as AD progressors, higher HOMA-IR predicted higher FDG in the medial temporal lobes and hippocampus, cerebral areas of interest in AD pathology [51]. Taken together, this evidence might support the use of insulin resistance as a marker, even in non-diabetes patients, to estimate the risk of progression from MCI to AD and to identify glucose metabolism patterns in MCI and AD.

The recent and growing effort to recognize cognitive impairment at the earliest possible phases has led to a fairly new definition of subjective cognitive decline (SCD); this may be an early stage of AD that precedes amnestic mild cognitive impairment. Some authors described metabolic pathways in “SCD plus” subjects, namely, those SCD subjects at high risk of progressing to MCI and AD, using magnetic resonance spectroscopy and metabolomics [52]. It was observed that serum levels of α-glucose and β-glucose were the highest in the MCI group, intermediate in the SCD plus group, and the lowest in the elderly control group. The authors also consider that neurons display impaired ability to utilize glucose during the preclinical stage of AD. Consequently, the brain appears to send a fasting signal that leads to the use of glucogenic amino acids and gluconeogenesis for energy maintenance. It was found that the levels of several glucogenic amino acids (aspartate, methionine, and threonine) were decreased in “SCD plus” patients, and the levels of even more glucogenic amino acids and their derivatives (aspartate, arginine, glycine, lysine, methionine, threonine, 1-Methylhistidine, glutamate, and glutamine) were reduced in the MCI group.

Even valine and its intermediates have been associated with neurodegeneration, and there is also evidence of altered valine levels in MCI and AD patients [53]. A further amino acid that might have a role in neurodegeneration is glutamine, and glutamate metabolism impairment was associated with preclinical dementia [54]. 

Similarly, alterations in the metabolism of sphingomyelin, which is synthesized from phosphatidylcholine, were related to preclinical and prodromal AD. Deficiencies of phosphatidylcholine and sphingomyelin synthesis may be due to inadequate phosphocholine. Thus, serum phosphocholine has the potential for use as another biomarker for the early detection of AD, and the early correction of abnormal phosphocholine metabolism may be a possible approach for the early treatment of AD [55]. In summary, the “metabolic pattern” of energetic efficiency from glucose and amino acids might represent a key for the early identification of SCD, MCI, and AD through non-invasive techniques, such as magnetic resonance spectroscopy and metabolomics.

A further hallmark of neurodegeneration in AD that has been largely demonstrated is the metabolic rewiring of pathways for glucose utilization and energy production. The human brain utilizes 20% of the total energy of the body. Glucose metabolism in the brain might differ in different types of cells. It has been demonstrated that neurons rely on oxidative phosphorylation for energy production, whereas astrocytes mostly utilize glycolysis [56]. The accumulation of β-amyloids in the aging brain has been shown to induce metabolic reprogramming similar to what is observed in cancerous cells. Tumour cells rely on aerobic glycolysis and concomitantly produce a large amount of lactate [57]; this metabolic shift is known as the “Warburg effect”. In healthy cells, the Krebs cycle, also known as the tricarboxylic acid cycle (TCA cycle), is the central metabolic pathway providing biochemical precursors for energy production. There is evidence that β-amyloids cause a shift from oxidative phosphorylation to aerobic glycolysis in both neurons and microglia [58].

This Warburg-like effect is evident even at early-stage AD. β-amyloid plaques interact with mitochondrial proteins, such as the complex IV cytochrome c oxidase, leading to impaired efficiency in oxidative phosphorylation [59]. Therefore, in early AD, aerobic glycolysis might be a neuroprotective compensatory response. However, chronic enhanced glycolysis leads to lactate accumulation, neuroinflammation, and increased apoptosis. In healthy neurons, glucose is metabolized through either the glycolysis or pentose phosphate pathway, followed by the Krebs cycle, and the oxidative phosphorylation system, resulting in the production of water, CO, and ATP molecules. The glycolysis process converts glucose to pyruvate, and then pyruvate is transported to the mitochondria in which pyruvate is used to synthesize acetyl-CoA. Further, acetyl-CoA is combined with citrate, which then enters the Krebs cycle. In Alzheimer’s disease, this “metabolic rewiring” in which pyruvate produced by glycolysis is largely converted into lactate has been associated with a particular isoform of neuronal pyruvate kinase, the pyruvate kinase M2 (PKM2). Some authors defined the PKM2 as a “metabolic signature” of glucose metabolism rewiring in AD. Neurons expressing PKM2 favour glycolysis despite a normal mitochondrial function, resembling the Warburg effect [60]. 

In humans, the pyruvate kinase M1 (PKM1) is ubiquitously expressed in adult differentiated tissues, including the brain, while PKM2 is usually expressed predominantly in the fetus, adult stem cells, and neoplastic tissues [61]. Unlike PKM1, PKM2 can be translocated into the cell nuclei and trigger the activation of transcription factors, such as the signal transducer and activator of transcription STAT3 and hypoxia-inducible factor 1α (HIF1α), ultimately modulating apoptosis [62]. PKM2 facilitates a Warburg-like glycolytic reprogramming in Alzheimer’s disease and increases neuron vulnerability to apoptosis. In later stages, AD is characterized by extensive neuronal cell death. This is particularly relevant because differentiated neurons in the healthy brain display efficient anti-apoptotic strategies to avoid neuronal loss. Conversely, neuronal de-differentiation might increase vulnerability to apoptosis and contribute to AD pathophysiology [63]. Neuronal PKM2 causes a metabolic shift and enhances neuronal apoptosis, which could be partially ameliorated with molecules acting as PKM2 modulators; furthermore, dietary intervention, such as the use of ketone-mediated nutritional therapeutics, might be a strategy to restore the metabolic imbalance caused by aerobic glycolysis in the neurons and microglia [64]. Like β-amyloids, tau phosphorylation negatively affects mitochondrial function, leading to reduced membrane potential, increased oxidative stress, and neurodegeneration in AD and other tauopathies [65]. Glial cells demonstrated similar metabolic rewiring induced by β-amyloids. Amyloid-activated microglia can enhance glycolysis in astrocytes via the AKT-mTOR-HIF-1α pathway, leading to increased lactate production and neuroinflammation [66].

Finally, in our review, we aim to examine the relationship between sarcopenia and cognitive impairment, two geriatric syndromes that frequently occur in clusters and seem to share common pathogenic mechanisms.

Sarcopenia is an age-related progressive and systemic loss of skeletal muscle mass, strength, and functional performance. It is associated with an increased risk of falls, disability, and frailty. There is mounting evidence of the association between sarcopenia and cognitive decline, even from heterogeneous demographics [67].

A relevant study investigated the associations of sarcopenia and its defining components with cognitive function in community-dwelling oldest old aged more than 80 years in China. The overall prevalence of sarcopenia was 35.5%, 40.34% for men and 32.14% for women. The prevalence of MCI was higher among the sarcopenic oldest old than the non-sarcopenic oldest old, and slow gait speed was significantly and independently associated with a risk of MCI [68].

Oudbier et al. hypothesize that myokines released by skeletal muscles are key regulators in the association between sarcopenia and cognitive decline [69]. Myokines affect neurogenesis, nervous system development, and neuroprotective pathways, and these pathways might be positively enhanced by physical activity. The myokine FNDC5 (fibronectin type III domain-containing protein 5) is a membrane protein primarily expressed in the muscle, brain, and adipose tissues. In mice animal models, endurance exercise enhances hippocampal BDNF expression via the PGC-1α-FNDC5-irisin pathway [70]; the cleaved peptide product of FNDC5, irisin, promotes neuronal proliferation and differentiation. Furthermore, irisin contributes to the neuroprotective effect of exercise via the activation of the AKT and ERK1/2 signalling pathways [71]. Physical exercise can induce PGC1-α expression and enhance the skeletal muscle secretion of myokines such as LIF, irisin, musclin, IGF-1, and BDNF [72]. BDNF also exerts its beneficial effect on mitochondria by activating AMP-activated protein kinases and enhancing fatty acid oxidation [73].

In conclusion, there is preliminary mounting evidence that sarcopenia induces alterations in myokine secretion, inflammation, lower peripheral glucose storage, and, ultimately, impaired cognition, but this muscle–brain connection and its role in cognitive impairment pathogenesis shall be further explored. 

Since sarcopenia is highly prevalent in those with dementia, sarcopenia should always prompt a comprehensive geriatric assessment in order to early identify SCD, MCI, or cognitive frailty. These measures may facilitate the earlier identification of sarcopenia and MCI, delaying disease progression, improving the quality of life of the elderly, and reducing social and economic burdens.

## 4. Conclusions

The crosstalk of metabolic dysregulation, neurodegenerative processes, and sarcopenia emphasizes the critical role of metabolic health in cognitive aging. Insulin resistance, obesity, and dyslipidemia not only contribute to systemic metabolic syndrome but also accelerate neurodegeneration, increasing the risk of cognitive impairment from SCD to MCI and AD.

Some cellular metabolic sensors, such as SIRT1, act as a critical regulator in aging, neuroprotection, and metabolic health, playing a pivotal role in cellular energy efficiency, DNA repair, and inflammation. Its activation, driven by calorie restriction, stress, nutraceutical, and pharmacological agents, enhances neuronal survival and mitigates neurodegenerative pathways in Alzheimer’s disease. Additionally, SIRT1 improves insulin sensitivity by silencing PTP1B expression, a negative regulator of insulin signalling, contributing to reduced adipose tissue and restoring insulin sensitivity. These findings might foresee the future therapeutic potential of SIRT1 activators in neurodegenerative and metabolic disorders, although large-scale studies are needed to confirm efficacy and safety in clinical settings.

As evidenced by both preclinical and clinical studies, interventions that address insulin resistance, such as GLP-1 receptor agonists, metformin, intermittent energy restriction, or fasting-mimicking agents, might target and mitigate neurodegeneration.

Mounting evidence highlights the potential of metabolic biomarkers, including triglyceride/HDL ratios, ApoA1 levels, and glucose metabolism patterns, on PET scans as predictive tools for cognitive decline. The association between sarcopenia, cognitive impairment, and impaired fasting glucose in the aging population reveals shared pathogenic molecular mechanisms and highlights the relevance of comprehensive geriatric assessment as a tool to identify clusters of aging-related disease.

## Figures and Tables

**Table 1:** Compounds with SIRT1 inducer activity.

Compound	Mechanism of Action	Effects
Resveratrol [11,12]	Activates SIRT1.	Improves cognitive function; protects against neurodegeneration.
Metformin [15]	Activates SIRT1 by increasing NAD+ levels; modulates AMPK enhancing SIRT1 activity indirectly.	Promotes mitochondrial biogenesis; improves insulin sensitivity.
Olmesartan [16]	Increases SIRT1 mRNA by inhibiting the angiotensin II type 1 receptor.	Reduces oxidative stress and inflammation.
SRT2104 [16]	Binds to an allosteric site on SIRT1, enhancing affinity for acetylated lysine residues on targets like p53, NF-kB, and PGC-1α.	Promotes deacetylation of substrates; improves metabolic and inflammatory responses without affecting NAD+ levels.
Curcumin [19]	Indirectly activates SIRT1 by scavenging ROS and inhibiting NF kB; binds directly to the SIRT1 active site.	Protects mitochondria; enhances oxidative phosphorylation.
Quercetin [20]	Activates SIRT1 through the AMPK; reduces ROS; inhibits mTOR; and increases NAD+ levels.	Promotes mitochondrial biogenesis; prevents β-amyloid-induced microglia death; reduces oxidative stress.
Pterostilbene [22]	Directly binds to SIRT1, enhancing deacetylase activity; increases NAD+ levels; and inhibits NAD+-consuming enzymes (CD38).	Reduces tau protein acetylation; prevents β-amyloid aggregation; supports cognitive function.
Nicotinamide Riboside [25,26,27]	Precursor for NAD+ biosynthesis.	Enhances NAD+ levels; promotes cellular energy metabolism and repair.

## Data Availability

No new data were created or analyzed in this study. Data sharing is not applicable to this article.

## Abbreviations

The following abbreviations are used in this manuscript:

GLP-1	Glucagon-Like Peptide 1
SCD	Subjective Cognitive Decline
IRS-1	Insulin Receptor Substrate 1
AD	Alzheimer’s Disease
BDGF	Brain-Derived Growth Factor
PCG	Peroxisome Proliferator-Activated Receptor Gamma Coactivator
NAD	Nicotinamide Adenine Dinucleotide
NF kB	Nuclear Factor Kappa Light-Chain Enhancer of Activated B Cells
FOXO	Fork Head Box O
PPAR	Peroxisome Proliferator-Activated Receptor
PTP1B	Protein Tyrosine Phosphatase 1B
AMPK	AMP-Activated Protein Kinase
ROS	Reactive Oxygen Species
MCI	Mild Cognitive Impairment
MPO	Myeloperoxidase
HOMA IR	Homeostatic Model Assessment of Insulin Resistance
FDG	Fluorodeoxyglucose
IGF-1	Insulin-Like Growth Factor 1
